# The psychology of manipulative visibility in the digital public sphere: a discourse analysis of audience reactions to deceptive Instagram content

**DOI:** 10.3389/fpsyg.2026.1848882

**Published:** 2026-08-18

**Authors:** Emel Yılmaz

**Affiliations:** Faculty of Fine Arts, American University of Cyprus, Lefkosa, Türkiye

**Keywords:** algorithmic visibility, authenticity, digital deception, digital public sphere, discourse analysis, Instagram reels, manipulative visibility, media psychology

## Abstract

**Introduction:**

The digital public sphere has become a primary site for social learning and belief formation, yet the psychological mechanisms through which manipulative content gains social acceptance remain underexplored from an educational psychology perspective. This study examines how visibility mechanisms on social media may function as informal learning environments that replace rational evaluation with heuristic processing and promote emotional, spectacle-driven participation. Drawing on theories of critical thinking development and sociocognitive learning, the study investigates how exposure to deceptive digital content shapes learners’ epistemic beliefs and evaluative standards. Analyzing a viral Instagram Reel by Turkish Show TV from 2021, titled “Virtual World, Fake World,” where a middle-aged man used FaceApp to appear as a young female motorcyclist, this research explores how deceptive content is created, spread, and normalized. The case is compared with international coverage from Vice, NDTV, and The Washington Post.

**Methods:**

The study employs an abductive approach in which empirical patterns are identified through inductive coding and interpreted in light of media psychology and educational theory, including Habermas’s analysis of the transformation of the public sphere. A total of 581 user comments were analyzed using thematic analysis to identify rhetorical strategies, emotional framing, and ideological interpretations. The analytical framework integrates perspectives on media literacy, critical thinking dispositions, and the role of affect in information processing to examine how audiences construct meaning from deceptive content.

**Results:**

The analysis reveals three recurring patterns in audience interpretations: (1) authenticity is reframed as entertainment rather than truth, suggesting a shift in evaluative criteria among digital learners; (2) humor reduces ethical concerns and normalizes deception, indicating that emotional engagement may override moral reasoning and critical evaluation; and (3) user participation is driven by visibility and emotion rather than deliberative debate, reflecting platform-conditioned learning behaviors that prioritize engagement over accuracy. These patterns suggest that algorithmic visibility and platform affordances shape the cognitive and affective dimensions of public communication, fostering participatory norms that may undermine the development of critical media literacy competencies.

**Discussion:**

The study contributes to media psychology and digital communication research by illustrating how deceptive content can become socially acceptable through visibility-based interaction. From an educational psychology standpoint, the findings underscore the need for pedagogical interventions that strengthen critical thinking skills, epistemic vigilance, and media literacy—particularly in informal learning contexts where platform algorithms serve as implicit curators of knowledge. As a single-case study selected for analytical richness rather than statistical representativeness, the findings are not intended to be generalized across all platforms or cultural contexts. Nevertheless, the study highlights the tension between ideal public-sphere values and platform-driven communication, emphasizing the need for enhanced media literacy education, scaffolded critical evaluation training, and platform regulations to mitigate the psychological impacts of manipulative digital narratives.

## 1 Introduction

Habermas’s work provides an important normative reference point for understanding these transformations. In his original formulation of the public sphere, communicative legitimacy emerged through rational-critical debate among citizens (Habermas, 2003). In his more recent reflections on the structural transformation of the public sphere, Habermas (2003, 2023) argues that digital communication environments have altered the conditions under which public opinion is formed by fragmenting audiences, expanding participation, and reshaping the relationship between communication and democratic will-formation. Rather than replacing Habermas’s framework, this study uses it as a critical benchmark for assessing contemporary forms of platform-mediated participation.

Castells (2013, 2020) concept of the network society and contemporary analyses of datafication and surveillance capitalism (Couldry and Mejias, 2019; Zuboff, 2019) further illuminate how visibility and platform architectures shape public communication. Methodologically, van Dijk (2008) Critical Discourse Analysis provides a framework for tracing how power and ideology circulate beneath the surface of open and participatory communication.

Building on these theoretical perspectives, the present study develops a preliminary conceptual framework for understanding manipulative visibility in the digital public sphere. The framework emerges abductively from the interaction between empirical findings and existing scholarship rather than from deductive theory testing. Its purpose is not to establish a new general theory of digital communication, but to provide an analytical lens through which the normalization of deception, visibility, and emotional engagement can be examined (Akşit, 2009).

From this perspective, visibility functions as an important mechanism through which content gains attention, legitimacy, and circulation within platform environments. The study explores how deceptive practices may become normalized through humor, emotional engagement, and algorithmically mediated interactions (Natale, 2024; Farid, 2025; Kim and Durcikova, 2025). Rather than claiming a distinct theory of the digital public sphere, the study offers a conceptual contribution that may support future comparative and theory-building research on digital communication.

To examine this framework, the analysis focuses on the circulation of a 2021 Instagram Reel published by Turkey’s Show TV titled “Virtual World, Fake World.” The study compares the discursive framing of this case with international coverage by Vice, NDTV, and The Washington Post and analyzes 581 user comments to explore how authenticity, deception, and visibility are negotiated through language, humor, and emotional engagement. In doing so, the study contributes both empirically and conceptually by proposing manipulative visibility as an analytical lens for examining contemporary forms of digital public communication.

## 2 Theoretical framework

While Habermas (2003, 2023) and Habermas (2022) conceptualizes the public sphere as a communicative space grounded in rational-critical exchange, contemporary media dynamics deviate from this normative ideal. Habermas’s more recent reflections on the structural transformation of the public sphere (2023) suggest that digital communication environments have altered the conditions under which public opinion is formed by expanding participation while simultaneously fragmenting audiences and attention.

Instead of substituting Habermas’s framework, this study uses his wider intellectual journey—from the historical public sphere to modern digital changes—as a guide to analyze how digital discourse varies from deliberative communication.

Building on these theoretical perspectives, this study creates an initial conceptual framework to analyze manipulative visibility in the digital public sphere. This framework results from an abductive dialog between empirical data and existing research, rather than from deductive theory testing. Its aim is not to establish a new comprehensive theory of digital communication but to serve as an analytical tool for examining how deception, visibility, and emotional engagement become normalized.

From a media psychology standpoint, this framework indicates that visibility could serve as a key organizational principle in today’s digital spaces, shaping the ways meaning, legitimacy, and inclusion are formed. Deceptive tactics might become normalized via humor, emotional connection, and algorithm-driven sharing. As a result, digital public communication may function as a performative arena where visibility plays a crucial role in how meaning is created, negotiated, and spread.

To situate the present study within recent scholarship on digital deception, algorithmic visibility, and media manipulation, Table 1 provides an overview of comparable research conducted in the last 5 years.

**Table 1:** Overview of recent research relevant to manipulative visibility.

Authors (Year)	Focus	Main finding	Relevance to current study
Kim and Durcikova (2025)	Deepfake normalization	Repeated exposure leads to desensitization and moral detachment	Supports discussion of normalization processes
Natale (2024)	Digital deception in everyday communication	Deception becomes embedded in routine digital practices	Supports normalization through everyday interaction
Aïmeur et al. (2023)	Misinformation and platform dynamics	Misinformation is culturally reproduced through platform structures	Demonstrates the role of platform dynamics in shaping discourse
Mulcahy et al. (2025)	Influencers and misinformation virality	Visibility-based engagement accelerates circulation	Supports visibility-driven engagement mechanisms
Farid (2025)	Deepfakes and manipulated media	Reviews strategies for reducing harms of manipulated content	Provides technical context for digital deception
Weikmann et al. (2025)	Deepfake exposure and credibility	Exposure reduces media credibility and increases AI-related anxiety	Illustrates consequences of deceptive content
Bakir et al. (2024)	Emotional responses to AI manipulation	Younger users show greater acceptance of manipulation technologies	Highlights demographic differences in perception
	Deepfake awareness and accountability	Entertainment framing reduces perceived responsibility	Supports discussion of ethical disengagement
Vaccari and Chadwick (2020)	Deepfakes and trust	Deepfakes undermine trust in news media	Supports discussion of credibility and trust
Dobber et al. (2021)	Deepfakes and micro-targeting	Manipulated content can influence attitudes and opinions	Demonstrates persuasive potential of algorithmic visibility

The following propositions are exploratory in nature and are intended to guide interpretation rather than provide deductively testable hypotheses.

Proposition 1: Visibility may increasingly function as an important source of legitimacy in digital discourse.

Proposition 2: Deception may be normalized through humor and emotional interaction.

Proposition 3: User engagement is often shaped by visibility and attention dynamics rather than by critical evaluation.

Proposition 4: Algorithmic systems tend to amplify emotionally resonant content regardless of their factual status.

Proposition 5: Authenticity may increasingly be reconstructed through performative and spectacle-oriented practices.

## 3 Method

In line with the proposed conceptual framework of manipulative visibility, this method allows for a detailed analysis of how visibility-driven legitimacy and emotional engagement influence digital conversations. The study employs an abductive approach, where empirical patterns are initially derived inductively from the data and then interpreted using existing theoretical perspectives. This reduces the risk of viewing theory solely as a means of confirmation and instead treats it as a tool for *post-hoc* interpretation. The main assumption examined is that visibility might increasingly serve as a source of legitimacy in digital communication, sometimes surpassing concerns about accuracy.

The case was chosen for its richness and high visibility as an example of digital deception, not for representing typical Instagram content. This aligns with qualitative case-study strategies that value interpretive depth over statistical generalization. The innovative aspect of this methodology is its connection with the conceptual framework, shifting focus from deception as misinformation to a visibility-centered, emotionally driven communication practice. By adopting this perspective, the study not only shows how manipulation spreads but also investigates how digital engagement may help reproduce and normalize it.

Initial coding was conducted using an open coding procedure. Recurrent themes such as humor, authenticity, visibility, moral evaluation, and audience participation were identified and subsequently grouped into broader interpretive categories through iterative comparison. The coding framework developed through this iterative process is presented in Table 2.

**Table 2:** Coding framework used in the analysis.

Initial code	Broader interpretive category
Humor	Normalization of deception
Authenticity	Identity construction
Visibility	Engagement logic
Moral evaluation	Ethical framing
Audience participation	Participatory response

International media reports were analyzed using the same comparative framing criteria applied to the Show TV coverage, including narrative tone, representations of authenticity, attribution of responsibility, and evaluative language. This procedure provided a systematic basis for cross-media comparison.

To enhance analytical credibility, coding decisions were documented in an audit trail and periodically reviewed during peer debriefing. These measures were adopted to increase transparency and reduce the risk of purely subjective interpretation. Rather than testing predefined theoretical assumptions, the analysis allows empirical patterns to emerge from the data before being interpreted through relevant theoretical perspectives.

The study follows an abductive logic in which empirical patterns are first allowed to emerge inductively from the data and are subsequently interpreted through existing theoretical perspectives. This approach reduces the risk of treating theory as a confirmation lens and instead positions it as a *post-hoc* interpretive framework.

## 4 Results

### 4.1 Background of the study

In 2021, a Twitter/X account attracted widespread public attention by presenting its owner (@azusagakuyuki) as a young female motorcyclist. The account was later revealed to belong to a 50-year-old man who used FaceApp after a follower noticed the reflection of a male figure in a motorcycle mirror. International media outlets such as Vice, NDTV, and The Washington Post covered the incident with relatively neutral, descriptive narratives, primarily emphasizing questions of authenticity, identity, and privacy (Vice, 2021; NDTV, 2021; The Washington Post, 2021).

However, in Turkey, the story was reported by Show TV under the title ‘Virtual World, Fake World.’ Dramatic visuals and amplified audio framed the event as a deception or hoax. Screenshot of a social media video from Show Haber on Instagram with the Turkish title “VIRTUAL WORLD, FALSE WORLD!” (Show TV, 2021). The screenshot shows “a person holding a large photograph displaying a digitally altered face.” The number of likes, comments, and shares are shown on the right. The caption discusses a phenomenon that deceives everyone. Table 3 presents a systematic comparison of how this event was framed by Show TV and by selected international outlets (Vice, NDTV, and The Washington Post) across four key dimensions: problem definition, causal attribution, moral evaluation, and treatment recommendation. This contrast is analytically significant because it demonstrates how the same event can acquire different cultural meanings depending on media framing, discursive context, and platform-specific communication practices.

**Table 3:** Systematic framing comparison of Show TV and international coverage.

Dimension	Show TV (Turkey)	Vice (International)	NDTV (International)	The Washington post (International)
Problem definition	“Virtual World, Fake World”—framed as hoax/spectacle	“Scammed Twitter”—framed as identity deception	“Uses FaceApp to become female influencer”—framed as technology-enabled transformation	“Posing as female influencer reveals online deception”—framed as privacy/authenticity issue
Causal attribution	Technology + individual deceit	Individual manipulation	FaceApp technology	Social media culture
Moral evaluation	Entertainment/sensationalism; moral shock as spectacle	Neutral/informative; mild concern	Neutral/descriptive	Critical; privacy concerns
Treatment recommendation	None (spectacle consumption)	Implicit warning about online trust	None (informational)	Implicit call for platform awareness

From the perspective of the proposed conceptual framework of manipulative visibility, this divergence is not merely a stylistic difference but reflects different visibility regimes. As illustrated in Table 3, these divergent framing strategies produce distinct visibility regimes that shape how audiences perceive and emotionally engage with deceptive content.

In this sense, visibility may function not only as a form of presentation but also as a mechanism that shapes how deception is perceived, experienced, and interpreted in digital public communication.

The comparative framing analysis reveals that while international outlets employed informational framing anchored in authenticity and privacy concerns, Show TV transformed the event into an affective spectacle. This divergence is not merely stylistic but reflects distinct visibility regimes: informational framing anchors meaning through referential accuracy, whereas spectacle framing enhances emotional engagement and circulatory potential.

From the perspective of the proposed conceptual framework of manipulative visibility, these findings suggest that visibility may operate as a key mechanism through which different media actors construct and circulate meaning in digital public communication.

In this sense, visibility may function not only as a form of presentation but also as a mechanism that shapes how deception is perceived, experienced, and interpreted in digital public communication.

### 4.2 News text analysis (show TV, 2021)

From the perspective of the proposed conceptual framework of manipulative visibility, the findings suggest that deception is not merely condemned but may also be transformed into a visible and consumable spectacle in which moral judgment coexists with entertainment and increased circulation. Second screenshot of an Instagram reel from Show Haber showing a woman smiling beside a yellow sign with Japanese text. The headline in Turkish says, “Sanal dünya yalan dünya!” meaning “Virtual world, false world!” Engagement icons display 7,755 likes, 581 comments, and 85 shares. The caption below reads “Fenomen herkesi kandıralı 4 sene geçti…” suggesting a phenomenon that has deceived everyone. This screenshot of the Show TV Instagram Reel interface documented the textual and visual composition through which the news report constructs deception as a consumable spectacle.

### 4.3 Quantitative indicators and participation dynamics

The third screenshot, also found on Show TV’s Instagram account, shows a social media video featuring a split image of the same person before and after their digital transformation. The text at the top reads “Virtual world, a world of lies!” in Turkish, with Japanese text in the middle. Interaction icons indicate 7,755 likes and 581 comments. The fourth screenshot, featured in the “Unusual” section of the NDTV news site, mentions a Twitter user, @azusagakuyuki, who shares motorcycle photos with over 20,000 followers, and is titled “Popular Female Biker Turned Out to Be a 50-Year-Old Man.” The fifth screenshot in the article, however, shows The Washington Post reporting that a ‘beautiful’ female biker was actually a 50-year-old man using FaceApp. The sixth screenshot of a VICE article headline reading, “Young Female Japanese Biker Turns Out To Be 50-Year-Old Man With FaceApp,” published by Koh Ewe on March nineteenth, two thousand twenty-one, at one thirty-one a.m. Below, two photos show a person with long hair beside a red and white Yamaha motorcycle on the left, and on the right, the same person without filter effects, both wearing similar motorcycle jackets. Viewed through the proposed conceptual framework, these findings suggest that visibility may function as a source of legitimacy, sometimes independently of factual accuracy.

In this context, deception does not necessarily reduce interaction and may, under certain conditions, contribute to further circulation and engagement.

In this case, deceptive content appears to function less as a barrier to participation than as a potential catalyst for circulation, consistent with research emphasizing the role of engagement-driven platform dynamics (Mulcahy et al., 2025).

### 4.4 Critical discourse analysis of audience comments

According to the proposed conceptual framework, these patterns imply that humor and visibility could serve as key mechanisms in normalizing deception. Rather than consistently triggering ethical reflection, user interaction may transform manipulation into a participatory spectacle, where engagement can become more salient than critical evaluation (Natale, 2024; Kim and Durcikova, 2025).

Overall, the findings suggest that this case is more than an isolated example of digital deception. The digital public sphere appears to be shaped by humor, spectacle, and algorithmic visibility, creating an environment that seems participatory while operating according to the logic of attention.

In this context, visibility may not only structure interaction but also contribute to the reconstruction of authenticity as a performative and negotiable construct. This case illustrates the tension between Habermas (2003, 2023) and Habermas (2022) deliberative ideal of public communication and contemporary forms of visibility-driven participation.

These interpretations emerged inductively from recurring patterns identified in the comment dataset and were subsequently contextualized through the conceptual framework of manipulative visibility.

## 5 Discussion

The proposed conceptual framework of manipulative visibility contributes to ongoing discussions of digital public communication by highlighting how visibility, emotional engagement, and platform dynamics may influence the normalization of deception. Traditionally, studies on digital deception have been either technically focused (detection of deepfake technologies) or macro-politically oriented (misinformation and disinformation campaigns). However, in recent years, the normalization processes of deception and its functioning within the visibility economy have received increasing scholarly attention.

Natale (2024) argued that digital media environments normalize deception by embedding it into everyday communication practices. Natale’s concept of “banalization” resonates with the patterns identified in the present study and provides a useful framework for interpreting the normalization of deception within everyday digital communication.

Kim and Durcikova (2025) found that repeated exposure to deepfake content leads to desensitization and moral disengagement, thereby accelerating normalization, particularly among young adults. The pattern identified in this study as “normalization through humor” aligns with Kim and Durcikova’s emotion-cognition framework, indicating that normalization may occur not only through cognitive processes but also through emotional mechanisms.

The findings of this study are consistent with research on visibility-driven engagement and suggest that visibility may play an important role in shaping circulation patterns and perceptions of legitimacy within digital environments. The pattern identified in this study as “normalization through humor” aligns with Kim and Durcikova’s emotion-cognition framework, indicating that normalization may occur not only through cognitive processes but also through emotional mechanisms.

This study engages directly with recent applications of Habermas (2023) theory of the public sphere to digital communication. Habermas’s analysis of the new structural transformation of the public sphere highlights how digitalization alters the conditions of deliberative opinion formation. While Habermas continues to emphasize the normative importance of rational deliberation, the present findings suggest that visibility-oriented participation has become a prominent feature of contemporary digital communication environments.

Castells (2013, 2020) theory of the network society illustrates how visibility and access restructure power relations. This study draws on Castells’ concept of communication power to explore how algorithmically mediated visibility may operate as a contemporary form of symbolic influence.

The studies summarized in Table 1 suggest that recent research on digital deception and manipulative communication has developed along three interconnected directions: (1) normalization and desensitization processes, (2) algorithmic visibility and viral circulation dynamics, and (3) perceptions of trust, responsibility, and ethics. These themes provide the broader scholarly context within which the present study develops its conceptual framework of manipulative visibility.

## 6 Conclusion

The analysis shows that the boundary between authenticity and deception in the digital public sphere is not merely undermined but actively produced and reproduced through discourse. Although Show TV’s “Virtual World, Fake World” report presents itself as a revelation, it also constructs an emotional, engaging narrative that may contribute to the normalization of manipulation. In Habermas (2003, 2023) terms, the case illustrates tensions between rational-critical deliberation and visibility-driven forms of participation.

These findings are broadly consistent with the proposed conceptual framework of manipulative visibility in the digital public sphere. More specifically, the findings suggest that visibility may function as an important source of legitimacy, that deception may become normalized through humor and emotional discourse, and that engagement is often shaped by spectacle and interaction rather than critical deliberation. In this sense, manipulation appears less as an isolated anomaly and more as a recurring feature of contemporary digital communication practices (Natale, 2024; Farid, 2025).

This form of participation may undermine digital trust and limit democratic potential, creating a communication environment in which freedom and control coexist. Rather than serving solely as an indicator of reality, authenticity may function as a performative resource through which manipulation becomes interpretable as entertaining rather than problematic. The study contributes to media and communication research by proposing manipulative visibility as an analytical lens for examining contemporary forms of digital public communication. The findings illustrate how platform dynamics and user interaction may contribute to the normalization of deception, highlighting tensions between normative public-sphere ideals and platform-mediated forms of engagement.

### 6.1 Limitations

This study has several limitations that should be acknowledged. First, as a single-case study, the findings are not statistically generalizable to all Instagram content or cross-cultural contexts. The case was selected for its analytical richness (extreme/deviant-case sampling) rather than its representativeness. Second, the primary data were drawn from a single platform (Instagram Reels) and a single national media outlet (Show TV), limiting the ability to assess cross-platform or cross-cultural variations. Third, although coding procedures were applied systematically, the majority of the dataset was coded by a single researcher, which may introduce interpretive bias despite the audit trail and peer debriefing procedures.

Fourth, the comparative framing analysis of international media was limited to three outlets and did not include systematic quantitative content analysis. Future research should employ multi-case, cross-platform designs with larger coder teams to enhance generalizability and reliability.

### 6.2 Future research directions

This study brings together three interconnected themes—normalization and desensitization processes, algorithmic visibility and viral circulation dynamics, and perceptions of trust, responsibility, and ethics—under the concept of manipulative visibility. Rather than offering a definitive theory, it proposes a conceptual framework that may support future empirical and comparative research.

Recommendations for future research include:

1. Comparative cross-platform analysis: Multiple case studies on TikTok, YouTube Shorts, and Instagram Reels can be conducted to compare different visibility regimes across platforms.
2. Longitudinal normalization studies: Using the framework of Kim and Durcikova (2025), the long-term moral and epistemic effects of repeated exposure can be examined.
3. Intervention and training models: As highlighted by Farid (2025) and Weikmann et al. (2025), effective ways to raise awareness of manipulation should be developed.
4. Regulatory policy analysis: As highlighted by Habermas (2023), structural adjustments may be necessary to strengthen the democratic potential of the public sphere.

## Data Availability

The original contributions presented in the study are included in the article/supplementary material, further inquiries can be directed to the corresponding author.
